# Celecoxib reduces brain dopaminergic neuronaldysfunction, and improves sensorimotor behavioral performance in neonatal rats exposed to systemic lipopolysaccharide

**DOI:** 10.1186/1742-2094-10-45

**Published:** 2013-04-05

**Authors:** Asuka Kaizaki, Lu-Tai Tien, Yi Pang, Zhengwei Cai, Sachiko Tanaka, Satoshi Numazawa, Abhay J Bhatt, Lir-Wan Fan

**Affiliations:** 1 Department of Pediatrics, Division of Newborn Medicine, University of Mississippi Medical Center, Jackson, MS, 39216, USA; 2 School of Medicine, Fu Jen Catholic University, Xinzhuang Dist, New Taipei City, 24205, Taiwan; 3 Department of Pharmacology, Toxicology and Therapeutics, Division of Toxicology, School of Pharmacy, Showa University, Shingawa-ku, Tokyo, 142-8555, Japan

**Keywords:** Cyclooxygenase-2, Dopamine uptake, Substantia nigra, Microglia, Astrocyte

## Abstract

**Background:**

Cyclooxygenase-2 (COX-2) is induced in inflammatory cells in response to cytokines and pro-inflammatory molecules, suggesting that COX-2 has a role in the inflammatory process. The objective of the current study was to examine whether celecoxib, a selective COX-2 inhibitor, could ameliorate lipopolysaccharide (LPS)-induced brain inflammation, dopaminergic neuronal dysfunction and sensorimotor behavioral impairments.

**Methods:**

Intraperitoneal (i.p.) injection of LPS (2 mg/kg) was performed in rat pups on postnatal Day 5 (P5), and celecoxib (20 mg/kg) or vehicle was administered (i.p.) five minutes after LPS injection. Sensorimotor behavioral tests were carried out 24 h after LPS exposure, and brain injury was examined on P6.

**Results:**

Our results showed that LPS exposure resulted in impairment in sensorimotor behavioral performance and injury to brain dopaminergic neurons, as indicated by loss of tyrosine hydroxylase (TH) immunoreactivity, as well as decreases in mitochondria activity in the rat brain. LPS exposure also led to increases in the expression of α-synuclein and dopamine transporter proteins and enhanced [^3^H]dopamine uptake. Treatment with celecoxib significantly reduced LPS-induced sensorimotor behavioral disturbances and dopaminergic neuronal dysfunction. Celecoxib administration significantly attenuated LPS-induced increases in the numbers of activated microglia and astrocytes and in the concentration of IL-1β in the neonatal rat brain. The protective effect of celecoxib was also associated with an attenuation of LPS-induced COX-2+ cells, which were double labeled with TH + (dopaminergic neuron) or glial fibrillary acidic protein (GFAP) + (astrocyte) cells.

**Conclusion:**

Systemic LPS administration induced brain inflammatory responses in neonatal rats; these inflammatory responses included induction of COX-2 expression in TH neurons and astrocytes. Application of the COX-2 inhibitor celecoxib after LPS treatment attenuated the inflammatory response and improved LPS-induced impairment, both biochemically and behaviorally.

## Background

In humans, the timing of maternal infection during pregnancy appears to play a crucial role in the neurodevelopmental responses/outcomes of offspring, and late gestational infection has been reported to induce perseverative behavior, which is implicated in schizophrenia and autistic spectrum disorders [1]. Human fetal brain development at late gestation roughly corresponds to the early postnatal period in rats (postnatal Day 7 (P7) in rats equals roughly the time of birth in humans) [2]. Our recent studies have shown that neonatal exposure to lipopolysaccharide (LPS) through an intracerebral (i.c.) injection in the rat brain (at P5, relevant to human intrauterine infection in late gestation) can produce brain inflammation, nigrostriatal dopaminergic injury and neurobehavioral dysfunction [3-8]. LPS, a component of the cell wall in gram-negative bacteria, is responsible for most of the inflammatory effects of infection from gram-negative bacteria. LPS has been detected in the amniotic fluid [9]. Thus, it is possible that LPS may reach the fetal brain during maternal infection.

Microglia have been identified as the major LPS-responsive cells in the central nervous system (CNS) [10]. Activation of microglia plays a critical role in perinatal i.c. LPS-induced dopaminergic neuronal injury in the rat brain [4,5,7,8]. Interaction of microglial cells with apoptotic neurons has been reported to selectively promote cyclooxygenase-2 (COX-2) expression, and COX-2 may mediate microglial activation and may play a key role in amplifying the inflammatory response with toxic effects [11,12]. In the CNS, COX-2 may have a physiological role; however, COX-2 is induced in inflammatory cells in response to cytokines and pro-inflammatory molecules, suggesting that COX-2 has a role in the inflammatory processes [11,13]. COX-2 is primarily responsible for prostanoid production in acute and chronic inflammatory processes, and its inhibition leads to anti-inflammatory effects [11,13]. COX-2 has also been hypothesized to be involved in many neurodegenerative diseases, such as multiple sclerosis, amyotrophic lateral sclerosis, Parkinson’s disease, Creutzfeldt-Jakob disease and Alzheimer’s disease [13].

Celecoxib is a selective COX-2 inhibitor and the safest COX-2 inhibitor in terms of cardiovascular safety data [14]. The neuroprotective action of celecoxib has been observed in the LPS-induced nigrostriatal neurodegeneration [15] and 6-hydroxydopamine (6-OHDA)-induced progressive dopaminergic neuron degeneration in a rat model of Parkinson’s disease [16]. The objective of the present study was to investigate whether systemic LPS exposure (at P5) through an i.p. injection also induced central inflammation, brain dopaminergic neuronal injury and sensorimotor behavioral deficits in our neonatal rat model whether celecoxib offered protection against LPS-induced brain inflammation and dopaminergic neuronal injury and improved sensorimotor behavioral performance in neonatal rats.

## Methods

### Chemicals

Unless otherwise stated, all chemicals used in this study were purchased from Sigma (St. Louis, MO, USA). Monoclonal mouse antibodies against β-amyloid precursor protein (APP), glial fibrillary acidic protein (GFAP), and α-synuclein were purchased from Millipore (Billerica, MA, USA; APP and GFAP) and BD Bioscience (San Jose, CA, USA; α-synuclein). Polyclonal rabbit antibodies against tyrosine hydroxylase (TH) and ionized calcium binding adapter molecule 1 (Iba1) were obtained from Millipore and Wako Chemicals USA (Irvine, CA, USA), respectively. Polyclonal rat antibodies against dopamine transporter (DAT) and polyclonal goat antibodies against COX-2 were obtained from Santa Cruz Biotechnology (Santa Cruz, CA, USA). [^3^H]Dopamine ([^3^H]DA, specific activity 34.6 Ci/mmol) was purchased from PerkinElmer (Boston, MA, USA). Enzyme-linked immunosorbent assay (ELISA) kits for immunoassay of rat interleukin-1β (IL-1β) and tumor necrosis factor-α (TNFα) were purchased from R&D Systems (Minneapolis, MN, USA).

### Animals

Timed pregnant Sprague–Dawley rats arrived in the laboratory on Day 19 of gestation. Animals were maintained in a room with a 12-h light/dark cycle and at constant temperature (22 ± 2°C). The day of birth was defined as postnatal Day 0 (P0). After birth, the litter size was adjusted to 12 pups per litter to minimize the effects of litter size on body weight and brain size. All procedures for animal care were conducted in accordance with the National Institutes of Health Guide for the Care and Use of Laboratory Animals and were approved by the Institutional Animal Care and Use Committee at the University of Mississippi Medical Center. Every effort was made to minimize the number of animals used and their suffering.

### Animal treatment

Injection of LPS (2 mg/kg i.p., from *Escherichia coli*, serotype 055: B5) was performed in five-day-old Sprague–Dawley rat pups of both sexes. The control rats were injected with the same volume of sterile saline (0.1 mL). All animals survived the injection. Both LPS- and saline-injected animals were further divided into two groups: one received an i.p. injection of celecoxib (20 mg/kg), and the other group received an i.p. injection of vehicle. Celecoxib (20 mg/kg) was dissolved in 20% dimethyl sulfoxide (DMSO) in normal saline [17,18] and administered immediately after the LPS injection. Thirty rats (15 male and 15 female pups) from each group were used in the present study. Behavioral tests were conducted in 12 rats from each group from P5 to P6. Rats were sacrificed on P6. Twenty-four rats from each group were sacrificed by decapitation to collect fresh brain tissue for Western blot analysis (six rats for each group), determination of the mitochondrial complex I activity (six rats for each group), ELISA assay (six rats for each group), and [^3^H]DA uptake study (six rats for each group). Six additional rats from each group were sacrificed by transcardiac perfusion with normal saline followed by 4% paraformaldehyde for brain section preparation. Free-floating coronal brain sections of 40-μm thickness were prepared in a freezing microtome (Leica, SM 2000R, Wetzlar, Germany) for immunohistochemistry staining.

### Behavioral testing

Behavioral tests were performed as described by Fan *et al.*[4], with modifications. The developmental test battery that was used was based on previously documented tests for neurobehavioral toxicity [19,20]. Behavioral tests, including the righting reflex and negative geotaxis test, were performed for all rat pups from P5 to P6.

### Righting reflex

This test is believed to be a reflection of muscle strength and subcortical maturation [20,21]. Pups were placed on their backs, and the time required to turn over on all four feet and touch the platform was measured. The cut-off time was 60 s.

### Negative geotaxis

This test is believed to test reflex development, motor skills, vestibular labyrinth and cerebellar integration [19,20]. Rats were placed on a 15° incline with their heads pointing down the slope and had to turn to face upward and begin to crawl up the slope. Each pup was given three trials a day, and the time spent to make a turn of 180° upward was recorded. The cut-off time was 60 s.

### Immunohistochemistry

Brain injury was estimated based on the results of immunohistochemistry in consecutive brain sections prepared from rats sacrificed one day (P6) after LPS injection. For immunohistochemistry staining, primary antibodies were used in the following dilutions: TH and Iba1, 1:500; GFAP, 1:200; and APP and COX-2, 1:100. TH was used to detect dopaminergic neurons in the substantia nigra (SN). The amount of APP, a membrane-spanning glycoprotein, in normal axons and neurons is not enough to be detected, but the accumulation of APP can be detected as an early sign of axonal and neuronal lesions [22,23]. Microglia were detected using Iba1 immunostaining, which recognizes both resting and activated microglia. GFAP was used to detect astrocytes. COX-2 provided selective staining of inducible cyclooxygenase. Sections were incubated with primary antibodies at 4°C overnight and further incubated with fluorescence-conjugated secondary antibodies (Alexa Fluor 555, 1:500 or Alexa Fluor 488, 1:200; Jackson Immunoresearch, West Grove, PA, USA) for 1 h in the dark at room temperature. DAPI (4^′^,6-diamidino-2-phenylindole) (100 ng/mL) was used simultaneously to identify nuclei in the final visualization. Sections incubated in the absence of primary antibodies were used as negative controls. When double-labeling was required, primary antibodies from different hosts were used in combination with appropriate secondary antibodies, which were raised against the immunoglobulin from the corresponding host. The resulting sections were examined under a fluorescent microscope (BX60, Olympus America Inc., Center Valley, PA, USA) at appropriate wavelengths.

### Immunoblotting analysis

Protein expression of DAT and α-synuclein was determined in P6 rat brains by Western blotting according to the methods of Fan *et al.*[7,8] and Hadlock *et al.*[24], with modifications. One day after LPS injection (P6), brains were quickly removed, and tissues were frozen in liquid nitrogen and stored at −80°C. Tissues were homogenized in an extraction buffer (Biosource, Camarillo, CA, USA), and a mixture of protease inhibitors (Calbiochem, La Jolla, CA, USA) and 1 mM phenylmethylsulfonyl fluoride (PMSF) was added, accompanied by application of a Sonic Dismembrator (Fisher Scientific, Suwanee, GA, USA) three times for 10 s each. Protein levels of homogenates were determined by the Bradford method. The homogenates were diluted 1:2 (v/v) with Laemmli sample buffer. Equal quantities of protein (10 μg/10 μL) were loaded into each well of a 4% to 20% SDS-polyacrylamide gradient gel (MINI-PROTEAN TGX, 4 to 20%, Bio-Rad Laboratories, Hercules, CA, USA). The separated proteins were transferred electrophoretically to polyvinylidene difluoride (PVDF) membranes (Bio-Rad Laboratories) at 100 V for 1 h. The blots were incubated with a blocking solution containing 5% nonfat milk and 0.1% Tween-20 in Tris-buffered saline (TBS) for 1 h before incubation with the primary antibody (1:1,000) in the blocking solution overnight at 4°C. The blots were then incubated with peroxidase-conjugated antibodies in blocking solution (1:4,000) for 1 h at room temperature. Immunoreactivity was detected by the Enhanced Chemiluminescence Plus or Advanced ECL system (GE Healthcare, Piscataway, NJ, USA). Images were acquired with the Chemidoc MP Imaging System followed by quantification using Image Lab software (both from Bio-Rad Laboratories). To ensure that equal amounts of protein were applied to the immunoblot, the membranes were stripped with a stripping buffer (Thermo Scientific, Rockford, IL, USA) and re-probed for β-actin (1:4,000, Sigma) to normalize the results.

### Synaptosomal [^3^H]DA (dopamine) uptake

Uptake of [^3^H]DA was determined according to the methods of Hadlock *et al.*[24] and Nickell *et al.*[25], with modifications. One day after LPS injection (P6), brain tissues were homogenized in ice-cold 0.32 M sucrose (50 mM Tris buffer, pH 7.4) and centrifuged (800 × *g* for 12 minutes; 4°C). The supernatant (S1) was then centrifuged (20,000 × *g* for 15 minutes; 4°C), and the resulting pellets (P2, synaptosomes) were resuspended in ice-cold water at concentrations of 2 mg/mL to lyse the synaptosomal membranes. Synaptosomal fractions were chilled at 4°C until DA uptake experiments commenced. Assays were performed in duplicate with a final volume of 250 μL. Aliquots of 25 μL synaptosomal fractions (50 μg of P2 protein) were added to tubes containing assay buffer (126 mM NaCl, 4.8 mM KCl, 1.3 mM CaCl_2_, 16 mM NaH_2_PO_4_, 1.4 mM MgSO_4_, 11 mM glucose and 1 mM ascorbic acid, pH 7.4) and 1 μM pargyline and then incubated at 37°C for five minutes. Nonspecific uptake was determined in the presence of 10 μM nomifensine. Samples were placed on ice, and 25 μL of 0.1 μM [^3^H]DA (10 nM final concentration) was added to each tube, after which accumulation was permitted to proceed for five minutes at 37°C. DA concentration and time of uptake were chosen based on the reports by Hadlock *et al.*[24] and Nickell *et al.*[25]. The reaction was terminated by the addition of 250 μL ice-cold assay buffer and subsequent filtration, followed immediately by the washing two times of ice-cold assay buffer. Radioactivity retained by the filters was counted using a liquid scintillation counter (PerkinElmer). Nonspecific uptake, defined as DA uptake in the presence of 10 μM nomifensine, was subtracted from total uptake to define DAT-mediated specific uptake.

### Determination of mitochondrial complex I activity

Complex I activity was determined by a spectrophotometric assay based on the quantification of the rate of oxidation of the complex I substrate NADH to ubiquinone as described by Champy *et al.*[26] and Hoglinger *et al.*[27], with minor modifications. Brain tissues from each pup were collected at 6 or 24 h after LPS injection. The frozen brain tissue was homogenized mechanically, sonicated on ice in 10 mM Tris–HCl buffer (pH 7.2) containing 225 mM mannitol, 75 mM saccharose and 0.1 mM EDTA, and then centrifuged (600 × *g*) for 20 minutes at 4°C, to obtain post-nuclear supernatants. The optical density of the supernatants (40 μg sample protein) in 1 mL an assay mixture was spectrophotometrically recorded at a wavelength of 340 nm for 200 s at 37°C. The assay mixture was a potassium phosphate buffer (25 mM, pH 7.5) containing 2 mM potassium cyanide, 5 mM magnesium chloride, 2.5 mg/mL bovine serum albumin, 2 μM antimycin A, 100 μM decylubiquinone and 300 μM NADH. The proportion of NADH oxidation sensitive to an excess of rotenone (10 μM) was attributed to the activity of complex I. This procedure minimizes the dissociation of rotenone from complex I because of the use of small buffer volumes, maintenance at low temperatures, and rapid analysis. The specific activity (nmol NADH oxidation/min/mg protein) of complex I (NADH-ubiquinone oxidoreductase) was calculated using a molar extinction coefficient ε_340nm_ = 6.22 mM^-1^ cm^-1^[28]. Enzyme activities were expressed as nmol/min/mg of brain tissue. Complex I activity was calculated as follows: Complex I activity = (Rate (min^-1^)/ ε_340nm_ (6.22 mM^-1^ cm^-1^))/ 0.040 mg.

### Determination of IL-1β and TNFα protein by ELISA

Two major pro-inflammatory cytokines, IL-1β and TNFα, were determined by ELISA as previously described [6,29]. Briefly, brain tissues from each pup were collected 24 h after LPS injection, when the LPS-stimulated increase in inflammatory cytokines in the rat brain reached a peak value [30]. Brain tissues were homogenized by sonication in 1 mL ice-cold PBS (pH 7.2) and centrifuged at 12,000 × *g* for 20 minutes at 4°C. The supernatant was collected, and the protein concentration was determined by the Bradford method. ELISA was performed following the manufacturer’s instructions, and data were acquired using a 96-well plate reader (Bio-Tek Instruments, Inc., Winooski, VT, USA). The cytokine contents were expressed as pg cytokine/mg protein.

### Quantification of data and statistics

Brain sections at the bregma level and the midbrain sections at a level one-third rostral from the lambda to the bregma were used for determination of the most pathological changes. Most immunostaining data were quantified by the counting of positively stained cells. When the cellular boundary was not clearly separated, numbers of DAPI-stained nuclei from the superimposed images were counted as the cell number. Three digital microscopic images were randomly captured in each of the three sections, and the number of positively stained cells in the three images was counted and averaged (cells/mm^2^). The mean value of cell counts from three brain sections was used to represent one single brain. For convenience of comparison among the treatment groups, results were standardized as the average number of cells/mm^2^. APP or COX-2 staining was quantified using National Institutes of Health (NIH) image software to determine the percentage area containing APP- or COX-2-positive staining in the entire area of the captured image [31]. In response to LPS challenge, the number of Iba1+ microglia and GFAP + astrocytes increases, and the soma of these cells become larger. In addition to cell density, Iba1 or GFAP immunoreactivity was also quantified by calculating the percentage area of the whole image containing Iba1 or GFAP immunostaining [7,8].

The behavioral data were presented as the mean ± SEM and analyzed by one-way ANOVA followed by the Student-Newman-Keuls test. Data from immunostaining, immunoblotting analysis, [^3^H]DA uptake, mitochondrial complex I activity and ELISA assay were presented as the mean ± SEM and analyzed by one-way ANOVA followed by the Student-Newman-Keuls test. Results with a *P*-value of less than 0.05 were considered statistically significant.

## Results

### Celecoxib improved sensorimotor behavioral deficits induced by LPS exposure

**Figure 1:**
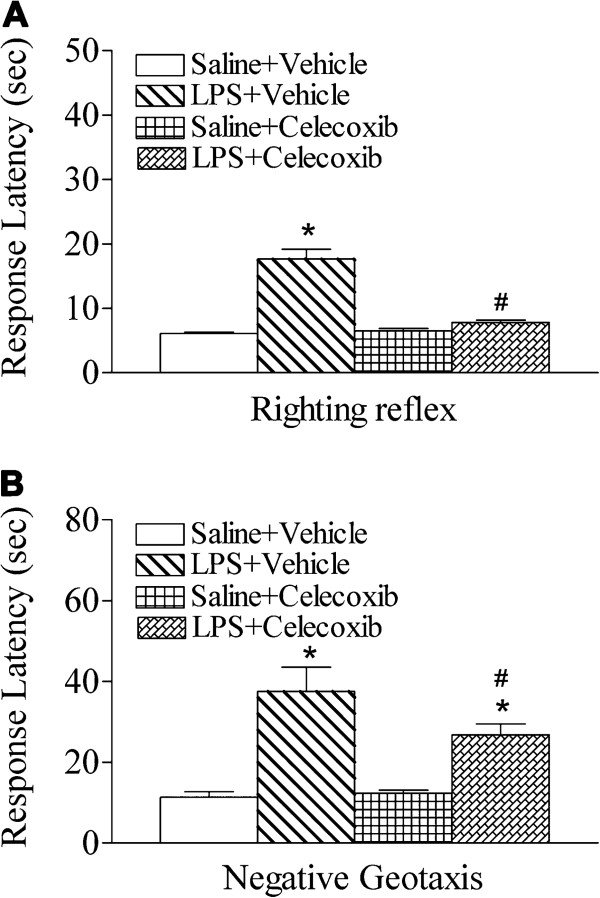
**Celecoxib attenuated systemic LPS-induced neurobehavioral deficits in the rat.** Celecoxib attenuated LPS-induced elongation of mean latency times in righting reflex (**A**) and negative geotaxis (**B**).

Compared with the control group, LPS-injection in P5 rats resulted in sensorimotor behavioral deficits at P6 (Figure 1A, B). Celecoxib treatment significantly improved sensorimotor behavioral performance following LPS exposure (Figure 1A, B).

### Righting reflex

The LPS-injected group exhibited significantly longer mean latency times as compared to the control group at P6 (*P* <0.05; Figure 1A). Celecoxib treatment significantly shortened the LPS-induced increase in righting reflex latency (*P* <0.05), and there was no difference in righting reflex between the control and the LPS + celecoxib groups (Figure 1A).

### Negative geotaxis

As shown in Figure 1B, the LPS-injected group exhibited significantly longer mean latency times for negative geotaxis along a 15° incline as compared to the control group at P6 (*P* <0.05). Celecoxib treatment significantly shortened the duration of the LPS-induced increase in negative geotaxis latency (*P* <0.05; Figure 1B).

### Celecoxib decreased LPS-induced dopaminergic neuronal and axonal damage

**Figure 2:**
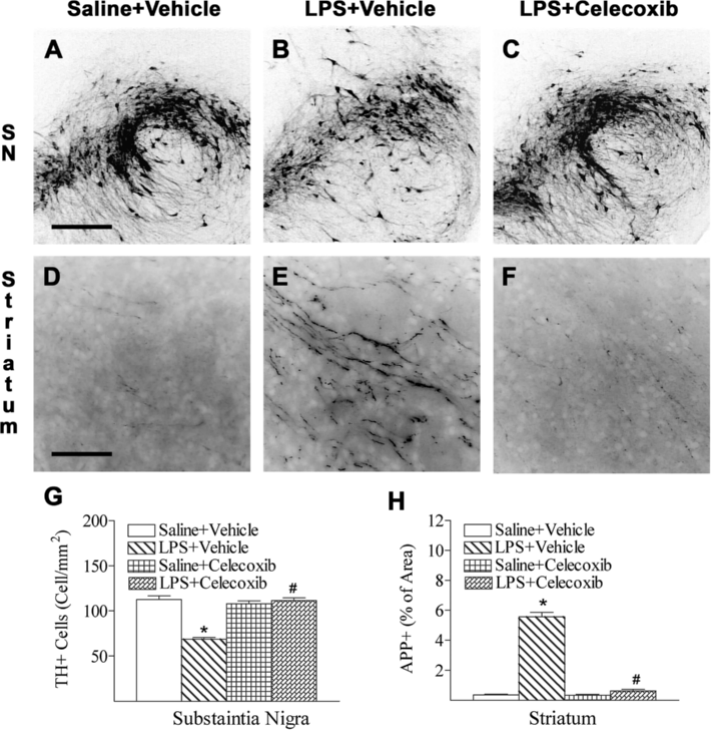
**Representative photomicrographs of TH (A to C, SN) and APP (D to F, striatum) immunostaining in the rat brain after LPS injection.** TH positive staining was detected in the SN area (**A**) of the midbrain sections at a level one-third rostral from the lambda to the bregma in the control rat brain. LPS injection caused a loss of TH positive staining (**B**). Celecoxib attenuated the LPS-induced loss of TH positive staining (**C**). Weak APP positive staining was detectable in the brain sections at the bregma level of the control brain (**D**). The beaded APP staining was found in the striatum (**E**) of the LPS-exposed brain. Celecoxib treatment attenuated the LPS-induced injury to axons in the striatum (**F**). The scale bar shown in **A** represents 200 μm for **A** to **C**, or shown in **D** represents 50 μm for **D** to **F**. Quantitation of the number of TH positive cells in the SN (**G**), and the percentage area of image that contained APP + staining in the cingulum white matter and the striatum were performed as described in Methods. The results are expressed as the mean ± SEM of six animals in each group, and analyzed by one-way ANOVA. *P <0.05 represents a significant difference for the LPS + Vehicle group or LPS + Celecoxib group as compared with the Saline + Vehicle group. ^#^*P* <0.05 represents a significant difference for the LPS + Celecoxib group as compared with the LPS + Vehicle group.

Positive staining for TH was used to detect dopaminergic neurons in the SN. In the P6 control rat brain, TH-positive cells were more predominant in the compact and lateral regions of the SN (Figure 2A, G). As shown in Figure 2B, neonatal systemic LPS exposure suppressed TH expression in the SN, as indicated by the reduced number of TH + neurons in the P6 rat brain (*P* <0.05; Figure 2B, G). This result was similar to the reduction of TH + staining induced by i.c. LPS injection that we reported previously [5]. Celecoxib treatment attenuated the LPS-induced reduction in the number of TH-positive neurons (*P* <0.05; Figure 2C, G).

Up-regulation of APP, an early sign of axonal and neuronal lesions [22,23], was determined by immunostaining in the striatum of P6 rats. As shown in Figure 2D, weak expression of APP was barely detectable in the control rat brain. Beaded APP immunostaining was observed in the LPS-exposed brain (*P* <0.05; Figure 2E, H). Celecoxib treatment attenuated the LPS-induced injury to axons in the striatum, as indicated by weak APP immunostaining, which was similar to that observed in control rats (*P* <0.05; Figure 2F, H).

### Celecoxib attenuated LPS-induced neuronal dysfunction and decreases in mitochondrial complex I activity

**Figure 3:**
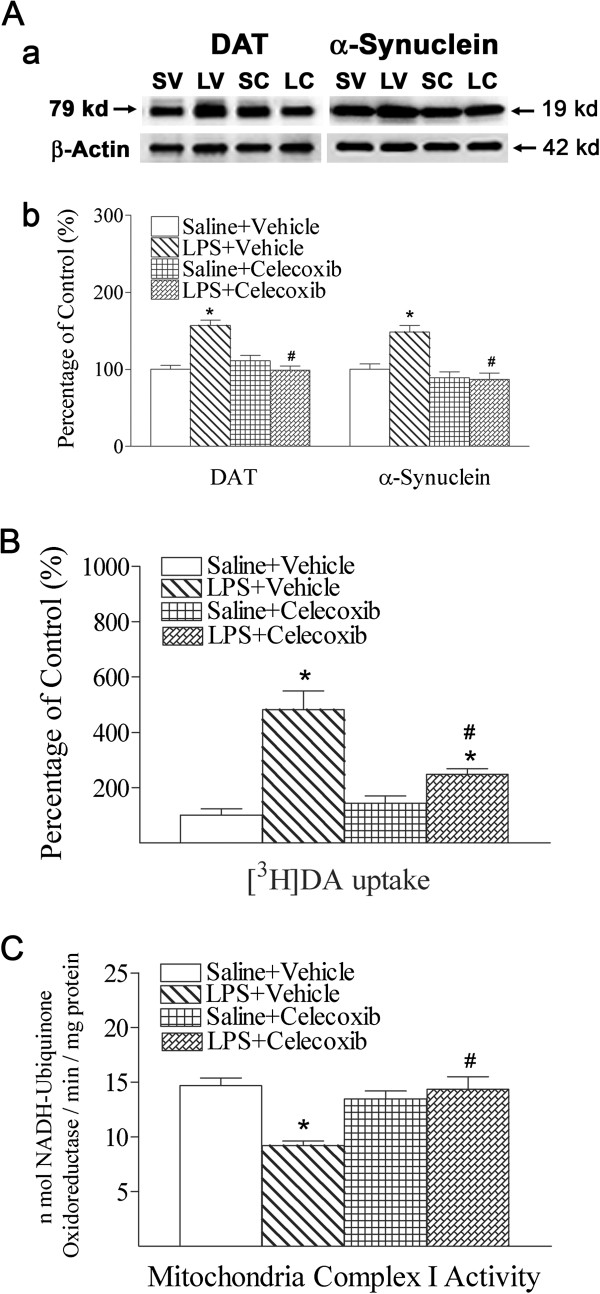
**Celecoxib attenuated the systemic LPS-induced change in expression of DAT and α-synuclein (A), [**^**3**^**H]DA uptake (B), and mitochondrial complex I enzymatic activity (C) in the rat brain.** Aa, Western blotting of protein expression of DAT and α-synuclein in P6 rat brain. Ab, Expression of DAT (left panel of Ab) and α-synuclein (right panel of Ab) is presented as the percentage of expression in the control group (Saline + Vehicle). Systemic LPS exposure increased DAT and α-synuclein expression in P6 rat brain. Celecoxib treatment attenuated the LPS-induced increase in expression of DAT and α-synuclein in the P6 rats. **B**, Dopamine uptake was measured by total [^3^H]DA uptake into brain synaptosomes and [^3^H]DA uptake is presented as the percentage of data in the control group (Saline + Vehicle). LPS exposure increased [^3^H]DA uptake in P6 rat brain. Celecoxib attenuated the LPS-induced increase in total [^3^H]DA uptake in the P6 rats. **C**, LPS exposure reduced enzymatic activity of mitochondrial complex I in 24 hours after LPS injection. Celecoxib treatment attenuated the LPS-induced decrease in mitochondrial complex I activity in P6 rats. The results are expressed as the mean ± SEM of six animals in each group, and analyzed by one-way ANOVA. *P <0.05 represents a significant difference for the LPS + Vehicle group or LPS + Celecoxib group as compared with the Saline + Vehicle group. ^#^*P* <0.05 represents a significant difference for the LPS + Celecoxib group as compared with the LPS + Vehicle group.

Neonatal systemic LPS exposure not only increased the amount of DAT and α-synuclein protein (*P* <0.05; Figure 3Aa, b), but also increased [^3^H]DA uptake (*P* <0.05; Figure 3B). Dopamine uptake was measured by total [^3^H]DA uptake into brain synaptosomes, as describe in the Methods section. These results revealed that LPS exposure may affect DAT function. Treatment with celecoxib significantly attenuated LPS-induced increases in the expression of α-synuclein and DAT proteins, as well as [^3^H]DA uptake (*P* <0.05; Figure 3A, B).

Mitochondrial complex I activity was measured as the amount of NADH oxidized per minute per milligram of protein in homogenates of whole brains of rats at 24 h after LPS injection. Systemic LPS exposure reduced enzymatic activity of mitochondrial complex I in 24 h (P6; *P* <0.05; Figure 3C). Celecoxib treatment attenuated the LPS-induced decrease in mitochondrial complex I activity in P6 rat brains (*P* <0.05; Figure 3C).

### Celecoxib decreased the LPS-induced increase in microglial activation and inflammatory responses

**Figure 4:**
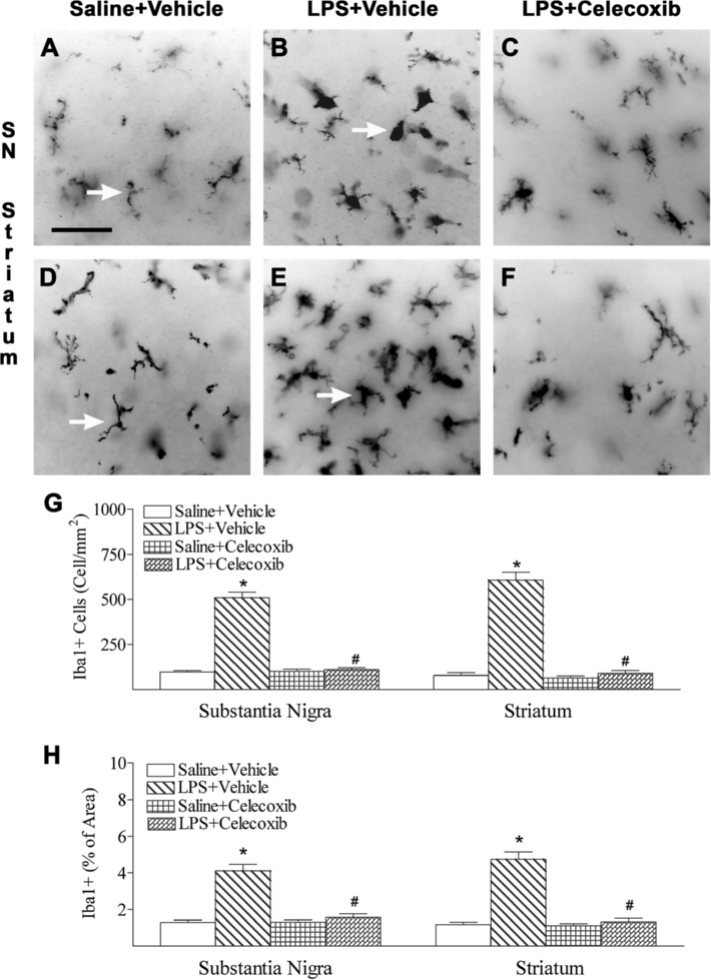
**Representative photomicrographs of microglia (A to C, SN; D to F, striatum) in the rat brain after LPS injection.** As shown by Iba1 immunostaining in the SN (**A**) and striatum (**D**), a few microglia at the resting status with a small rod-shaped soma and ramified processes (arrows indicated in **A** and **D**) were found in the control rat brain. Numerous activated microglia showing bright staining of an elongated or a round shaped cell body with blunt or no processes (arrows indicated in **B** and **E**) were observed in the SN (**B**) and striatum (**E**) of the rat brain with neonatal LPS exposure. Celecoxib treatment reduced the number of activated microglia stimulated by LPS in the above areas (**C**, **F** and **G**). The scale bar shown in A represents 50 μm for **A** to **F**. Quantitation of the number of Iba1+ cells (**G**) and the percentage area of image that contained Iba1 staining (H) in the SN and striatum were performed as described in Methods. The results are expressed as the mean ± SEM of six animals in each group, and analyzed by one-way ANOVA. *P <0.05 represents a significant difference for the LPS + Vehicle group as compared with the Saline + Vehicle group. ^#^*P* <0.05 represents a significant difference for the LPS + Celecoxib group as compared with the LPS + Vehicle group.

Activated microglia were assessed by Iba1 immunostaining in the rat SN (Figure 4A–C, G, H) and striatum (Figure 4D–H). LPS treatment triggered the activation of microglia in the SN (Figure 4B, G, H) and striatum (Figure 4E, G, H). In control rat brains, a few Iba1-positive cells were detected, and most of these cells were in a resting state with a ramified shape in both the SN and striatum (arrows indicated in Figure 4A, D). Significantly increased numbers of activated microglia (Figure 4G) showing bright staining of an elongated or a round-shaped cell body with blunt or no processes were found in the SN and striatum (arrows indicated in Figure 4B, E) 24 h after LPS injection (*P* <0.05). Iba1 staining was also quantified by measuring the percentage area containing Iba1 immunostaining in the captured images. Higher percentages of Iba1 immunostaining areas were observed in the SN and striatum of neonatal LPS-exposed rat brains (Figure 4H). Celecoxib treatment reduced the number of activated microglia and percentage of Iba1 immunostaining area following LPS injection (*P* <0.05; Figure 4C, F–H).

**Figure 5:**
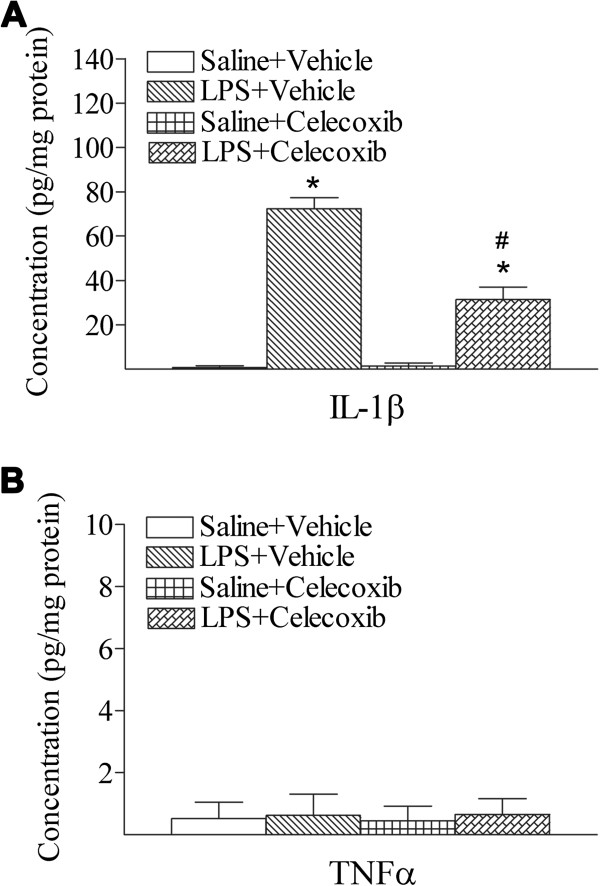
**Celecoxib attenuated systemic LPS-stimulated increases in inflammatory cytokine, IL-1β in the rat brain. A**, IL-1β and **B**, TNFα concentrations were determined by ELISA kit and presented in the unit of pg/mg protein, as described in Methods. Twenty-four hours following LPS injection, the brain level of IL-1β (A) was elevated as compared with the Saline + Vehicle group. Treatment with celecoxib attenuated induction of IL-1β content by LPS. The results are expressed as the mean ± SEM of six animals in each group, and analyzed by one-way ANOVA. * *P* <0.05 represents a significant difference for the LPS + Vehicle group or LPS + Celecoxib group as compared with the Saline + Vehicle group. ^#^*P* <0.05 represents a significant difference for the LPS + Celecoxib group as compared with the LPS + Vehicle group.

Systemic exposure to LPS resulted in inflammatory responses in the rat brain, as evidenced by the elevated expression of a major pro-inflammatory cytokine, IL-1β (Figure 5A). However, the concentration of TNFα in the rat brain returned to the control level 24 h after LPS exposure (Figure 5B). Treatment with celecoxib attenuated the induction of IL-1β content by LPS (*P* <0.05; Figure 5A).

### Celecoxib decreased the LPS-induced increase in astrocyte activation and COX-2 expression

**Figure 6:**
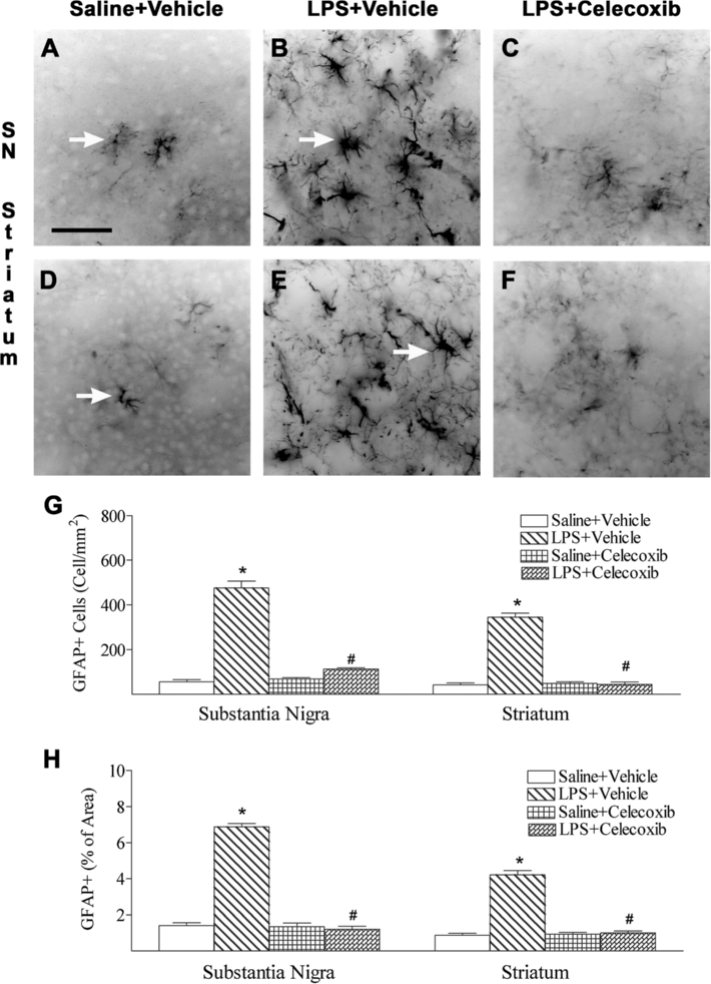
**Representative photomicrographs of astrocytes (A to C, SN; D to F, striatum) in the rat brain after LPS injection.** As shown by GFAP immunostaining in the SN (**A**) and striatum (**D**) in the control rat brain, some GFAP positive cells were detected and most of those cells were in resting status with fine processes extending from the main cellular processes (arrows indicated **A** and **D**). Significantly increased numbers of reactive astrocytes showing hypertrophy of cellular processes (arrows indicated in **B** and **E**) were observed in SN (**B**) and striatum (**E**) of the rat brain following neonatal LPS exposure. Celecoxib treatment reduced the number of reactive astrocytes stimulated by LPS in the above areas (**C**, **F** and **G**). The scale bar shown in A represents 50 μm for **A** to **F**. Quantitation of the number of GFAP + cells (**G**) and the percentage area of image that contained GFAP staining (**H**) in the SN and striatum were performed as described in Methods. The results are expressed as the mean ± SEM of six animals in each group, and analyzed by one-way ANOVA. *P <0.05 represents a significant difference for the LPS + Vehicle group as compared with the Saline + Vehicle group. ^#^*P* <0.05 represents a significant difference for the LPS + Celecoxib group as compared with the LPS + Vehicle group.

Development of hypertrophic morphology and up-regulation of intermediate filament proteins, such as GFAP, by reactive astrocytes are perhaps the best known hallmarks of reactive astrocytes and reactive gliosis [32,33]. Increased expression of GFAP, an indication of astrogliosis, was observed at the SN (Figure 6B) and striatum (Figure 6E) 24 h after injection in rats exposed to systemic LPS (Figure 6G). In control rat brains, some GFAP-positive cells were detected, and most of these cells were in the resting state, with fine processes extending from the main cellular processes (arrows indicated in Figure 6A, D). Significantly increased numbers of reactive astrocytes showing hypertrophy of cellular processes of astrocytes (GFAP + cells) were found in the SN (arrows indicated in Figure 6B) and striatum (arrows indicated in Figure 6E) of rat brains 24 h after LPS injection (*P* <0.05; Figure 6G). GFAP staining was also quantified by measuring the percent area containing GFAP immunostaining in the captured images. A higher percentage of GFAP immunostaining area was observed in the SN and striatum of neonatal LPS-exposed rat brains (Figure 6H). Celecoxib treatment reduced the number of activated astrocytes and the percentage of GFAP immunostaining area following LPS injection (*P* <0.05; Figure 6C, F–H).

**Figure 7:**
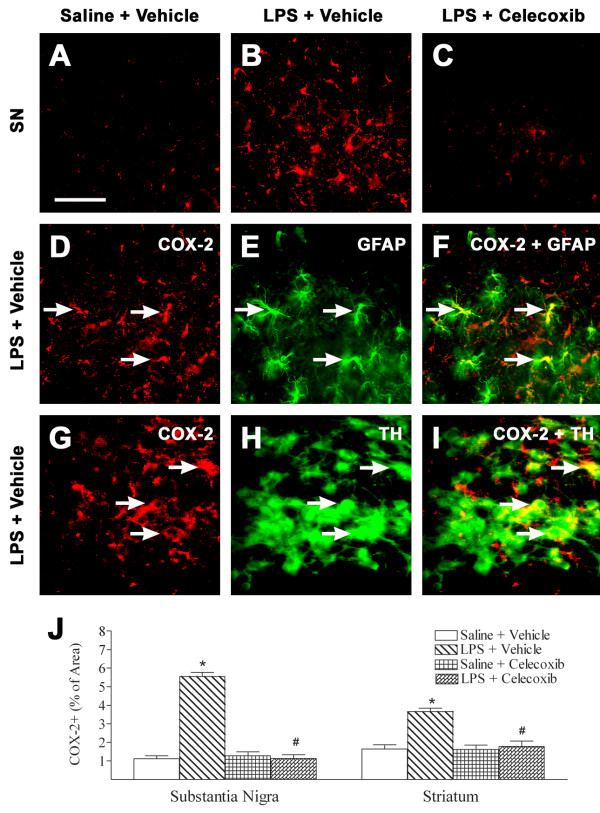
**Representative photomicrographs of COX-2 immunostaining (A to I, SN) in the rat brain after LPS injection.** Weak COX-2 positive staining was detectable in the brain sections of control brain (**A**). LPS injection increased the expression of inducible cyclooxygenase, as indicated by COX-2+ staining, in SN (**B**, **D** and **G**, red) and striatum (**J**) of P6 rat brain. Celecoxib treatment attenuated the LPS-increased COX-2+ staining in the above areas (**C** and **J**). F is a merged image of **D** and **E**. Double-labeling (yellow) showed that many COX-2 positive cells (**F**) were GFAP + cells (astrocytes) (**E**, green). I is a merged image of **G** and **H**. Double-labeling (yellow) also showed that some COX-2 positive cells (**I**) were TH + cells (dopaminergic neuron) (**H**, green). The scale bar in A represents 50 μm for **A** to **I**. Quantitation of the percentage area of image that contained COX-2+ staining in the SN and striatum was performed as described in Methods. The results are expressed as the mean ± SEM of six animals in each group, and analyzed by one-way ANOVA. *P <0.05 represents a significant difference for the LPS + Vehicle group as compared with the Saline + Vehicle group. ^#^*P* <0.05 represents a significant difference for the LPS + Celecoxib group as compared with the LPS + Vehicle group.

Neonatal systemic LPS-induced inflammatory responses were also observed, as indicated by the increase in the percent area containing COX-2+ cells in the rat SN (Figure 7B, D, G, J) and striatum (Figure 7J) as compared to that in the control rat SN (Figure 7A, J) and striatum (Figure 7J), respectively. Double staining showed that most COX-2+ cells in the SN were co-localized with GFAP + cells (Figure 7E, F), and some of these double-labeled cells were also co-localized with TH + neurons (Figure 7H, 7I). There were few COX-2+ cells that localized with Iba1-expressing microglia (data not shown). Treatment with celecoxib reduced the increase in percentage of COX-2 immunostaining area in the rat SN and striatum following LPS injection (*P* <0.05; Figure 7C, J).

## Discussion

Our results indicated that, similar to i.c. LPS injection, systemic exposure to LPS through i.p. injection in neonatal rats cause brain inflammatory responses and sensorimotor behavioral impairment, as well as damage to the dopaminergic system in the rat brain [3-6,29,34]. Neonatal systemic LPS exposure resulted in brain inflammatory responses in the rat, as indicated by an increased number of activated microglia (Figure 4) and elevated IL-1β concentrations in LPS-treated rat brains (Figure 5A). Our previous data also show that activation of microglia plays a critical role in perinatal i.c. LPS-induced dopaminergic neuronal injury in rat brains [4,5,8]. Microglia, the major resident immune cells in the brain, have been identified as the major LPS-responsive cells in the CNS [10]. Microglia are detectable in the CNS of early embryos, but the largest population of newborn microglia emerges in late gestation and the early postnatal period in both humans and rats [35,36]. Thus, the LPS exposure in perinatal rat brains (P5, relevant to human intrauterine infection in late gestation) can produce substantial inflammatory responses in the brain. LPS treatment also induced the expression of COX-2 in cells that were double labeled with TH + (dopaminergic neurons) or GFAP + (astrocytes) cells in neonatal rat brains (Figure 7). Interactions between microglial cells and apoptotic neurons have been reported to selectively promote COX-2 expression, and COX-2 may mediate microglial activation and may play a key role in amplifying the inflammatory response with toxic effects [11,12]. The current study showed that treatment with a selective COX-2 inhibitor, celecoxib, elicited anti-inflammatory effects, as evidenced by the attenuation of LPS-induced increases in the number of activated microglia and in the concentration of IL-1β in neonatal rat brains.

Increased expression of GFAP, an indicator of astrogliosis, was observed in the SN and striatum in rats 24 h after systemic LPS exposure (Figure 6). Treatment with celecoxib affected LPS-induced astrogliosis (Figure 6) and reduced the number of GFAP + and COX2+ double-labeled cells in LPS-exposed rat brains (Figure 7). Reactive astrocytes usually do not attack pathological targets, as do microglia, but instead wall off such targets to form a syncytium of interconnected cells, both in healthy and diseased states [32,33]. Astrocytes produce both pro-inflammatory and anti-inflammatory responses; for example, astrocytes may stimulate the microglia and secrete protective factors to the peripheral region at the same time [32]. α-Synuclein has been shown to activate both microglia and astrocytes, and these interactions may contribute to dopamine quinine formation [37]. However, the precise mechanisms of interaction between astrogliosis and dopaminergic neuronal injury are unclear, and further studies are needed.

Neonatal systemic LPS exposure resulted in dopaminergic system disturbances, as indicated by sensorimotor impairments, a decrease in the number of TH + cells in SN, and increases in the expression of α-synuclein and DAT proteins, and an increase in [^3^H]DA uptake in rat brains (Figures 1, 2, 3). Neuroinflammation and α-synuclein dysfunction have been proposed to potentiate each other; this may drive chronic progression of neurodegeneration [38]. The present study also showed that LPS exposure induced COX-2 expression in dopaminergic neurons of the rat SN (Figure 7). Increased COX-2 expression in dopaminergic cells under stressful conditions can facilitate dopamine oxidation to quinone species, triggering oxidative stress, and COX-2 overexpression in dopaminergic cells may also play a role in α-synuclein accumulation [11,39]. Other reports have implied that α-synuclein may modulate DAT function, and disruption of this modulatory process may permit increased re-uptake of high levels of intracellular dopamine by DAT, causing profound neurotoxicity [40,41]. Moreover, α-synuclein can associate with the inner mitochondrial membrane and mitochondrial α-synuclein accumulation results in complex I impairment in dopaminergic neurons and increased free radical production [42]. The current study found that treatment with the COX-2 inhibitor celecoxib attenuated the LPS-induced decrease in mitochondria complex I activity, dopaminergic injury and dopaminergic dysfunction. Therefore, the neuroprotective effects of COX-2 inhibition may be related to the blockade of COX-2-mediated dopamine oxidation and the inhibition of amplification of the inflammatory response, causing toxic effects [11,12].

LPS may provoke a dramatic systemic response, including up-regulation of inflammatory mediators and procoagulant factors in the systemic circulation, and LPS may also cause diarrhea, changes in plasma protein binding capacity, and modulation of hepatic and/or intestinal microsomal cytochrome P450 (CYP) isozymes, thereby affecting the renal excretion of drugs [43,44]. Therefore, the central neuroprotective effect of celecoxib may result from not only direct COX-2 inhibition in the brain, but also the effects of celecoxib on peripheral inflammatory responses. Other reports have indicated that intracerebral administration of LPS in rodents induces strong increases in COX-2 expression, mainly in astroglia and microglia, whereas COX-1 expression was predominantly observed in microglia and did not increase [45]. However, it has been suggested that, owing to its predominant localization in microglia, COX-1 may be the major player in neuroinflammation, whereas COX-2, which is localized in neurons, may have a major role in models in which the neurons are directly challenged [46]. Therefore, COX-1 preferential inhibitors also need to be further investigated in neurodegenerative diseases.

COX-2 has been suggested to be associated with various inflammatory parameters and is thought to be involved in neurodegenerative processes, such as multiple sclerosis, amyotrophic lateral sclerosis, Parkinson’s disease, Creutzfeldt-Jakob disease and Alzheimer’s disease [11,13]. Celecoxib is a selective COX-2 inhibitor and has been shown to be the safest COX-2 inhibitor in terms of cardiovascular safety data [14]. The neuroprotective action of celecoxib has been observed in LPS-induced nigrostriatal neurodegeneration [15] and 6-hydroxydopamine (6-OHDA)-induced progressive dopaminergic neuron degeneration in a rat model of Parkinson’s disease [16]. Our present results also suggested that celecoxib may provide protection against systemic LPS exposure-induced dopaminergic neuronal dysfunction and sensorimotor behavioral disturbances; these protective effects are likely associated with its anti-inflammatory properties. However, the neuroprotective effects of celecoxib are still controversial since celecoxib has been reported to prevent LPS-induced cognitive impairments in mice [47], but also to worsen spatial memory retention in rats [48]. Epidemiologic evidence suggests that celecoxib may delay the onset of Alzheimer’s dementia [49], but there is no benefit from celecoxib in symptomatic Alzheimer’s disease [50]. Thus, the chronic use of celecoxib may be beneficial only in the very early stages of the Alzheimer’s disease process [51]. Detailed mechanisms of celecoxib involvement in reducing systemic LPS exposure-induced dopaminergic neuronal dysfunction and in protection against LPS-induced sensorimotor behavioral disturbances need further investigation.

## Conclusion

Brain inflammation induced through systemic LPS exposure is clinically relevant, and our current findings indicated that systemic LPS exposure (P5) through an i.p. injection induced central inflammation; these inflammatory responses included induction of COX-2 expression in TH neurons and astrocytes. Our results also suggested that application of the COX-2 inhibitor celecoxib after LPS injection can attenuate the inflammatory response and improve LPS-induced impairment, including dopaminergic neuronal dysfunction and sensorimotor behavioral disturbances. The current results provide valuable information for developing strategies in the prevention and therapeutic treatment of neurodegenerative diseases.

## Abbreviations

6-OHDA: 6-hydroxydopamine; APP: β-amyloid precursor protein; CNS: Central nervous system; COX-2: Cyclooxygenase-2; CYP: cytochrome P450; DA: Dopamine; DAT: Dopamine transporter; DAPI: 4′,6-diamidino-2-phenylindole; DMSO: Dimethyl sulfoxide; ELISA: Enzyme-linked immunosorbent assay; GFAP: Glial fibrillary acidic protein; Iba1: Ionized calcium binding adapter molecule 1; IL1-β: Interleukin 1-β; i.c: Intracerebral; i.p: Intraperitoneal; LPS: Lipopolysaccharide; P5: Postnatal day 5; PMSF: phenylmethylsulfonyl fluoride; PVDF: polyvinylidene difluoride; SN: Substantia nigra; TBS: Tris-buffered saline; TH: Tyrosine hydroxylase; TNFα: Tumor necrosis factor alpha

## Competing interests

The authors declare that they have no competing financial or personal interests, and that none of the authors’ institutions have contracts relating to this research through which it may stand to gain financially now or in the future.

## Authors’ contributions

AK established the protocols and carried out the experiments, and drafted the manuscript. LTT and YP performed the experiments and the statistical analysis. ST and SN participated in the data analysis and helped to draft the manuscript. AJB was involved in editing drafts of the manuscript. LWF and ZC designed the study, coordinated the experiments and co-wrote the manuscript. All authors read and approved the final manuscript.
